# It’s too quick to blame myself—the effects of fast and slow rates of change on credit assignment during object lifting

**DOI:** 10.3389/fnhum.2014.00554

**Published:** 2014-07-29

**Authors:** Kelene Fercho, Lee A. Baugh

**Affiliations:** ^1^Sanford School of Medicine, University of South DakotaVermillion, SD, USA; ^2^Center for Brain and Behavior Research, University of South DakotaVermillion, SD, USA

**Keywords:** credit assignment, object lifting, load force, rates of chage, motor learning

## Abstract

Although there have been substantial research efforts examining the effect of various rates of change in reaching movements, there has been little to no research devoted to this issue during object manipulation tasks. In force-field and visuomotor adaptation studies, two parallel processes have been identified: first, a fast process that adapts and de-adapts quickly is thought to enable the actor to deal with potentially transient perturbations. Second, a slower, but longer lasting process adapts if these initial perturbations persist over time. In a largely separate body of research, the role of credit assignment has been examined in terms of allotting the cause of errors to changes in the body vs. changes in the outside world. Of course, these two processes are usually linked within the real world, with short lasting perturbations most often being linked to external causes and longer lasting perturbations being linked to internal causes. Here, we demonstrate that the increases in load forces associated with a gradual increase in object weight during a natural object lifting task are transferred when lifting a novel object, whereas a sudden increase in object weight is not. We speculate that gradual rates of change in the weight of the object being lifted are attributed to the self, whereas fast rates of change are more likely to be attributed to the external environment. This study extends our knowledge of the multiple timescales involved in motor learning to a more natural object manipulation scenario, while concurrently providing support for the hypothesis that the multiple time scales involved in motor learning are tuned for different learning contexts.

## Introduction

Although our motor system is finely tuned to generate accurate movements when interacting with our environment, we inevitably make many mistakes on a daily basis when manipulating our surroundings. Luckily, the motor system is capable of adapting future movement based on the errors experienced in previous interactions with the world. To fully-benefit from this type of error-based learning, the underlying cause of experienced errors must be identified. For example, suppose you are playing a round of golf, and on the 10th hole your drive off the tee falls much shorter than you had predicted. When the swing is made, the sensorimotor system is capable of comparing the movement’s outcome to a desired and/or predicted state. The information that results from this comparison can be used to inform the motor system that the target goal was not attained, and provides some initial information as to how the target was missed. In our golf example, this error-based learning could be used to adjust the motor commands for the following swing. Error-based learning is well-understood, having been examined in many variants of adaptation paradigms including saccadic adaptation (Pélisson et al., 2010), reaching in force fields (Thoroughman and Shadmehr, 2000), and grip force modulation (Flanagan and Wing, 1997).

It is, therefore, apparent that our motor system is capable of adapting future movements based on errors experienced in previous scenarios. However, in the real world, there are a number of potential causes for a given motor movement that could result in an unexpected outcome. If we return to the golf example, a strong headwind or fatigue could both result in the shot falling shorter than expected, but the corrections the motor system should engage in would be different for each situation. Although the end goal of needing to hit the ball further is equivalent in both scenarios, the optimal way to achieve that goal is not. If a gust of wind is responsible, any changes in the motor plan related to the swing should be temporary. However, if general fatigue is to blame, motor plans should be adjusted for the remainder of the game. In order to maximize motor performance, assigning blame to the correct cause is essential, and is a credit assignment problem. Research has shown that when we learn new dynamics related to a movement, we are able to link learning to appropriate contextual cues. This, in turn, allows for the cause of any errors to be linked to the self, vs. the external world. For instance, after-effects, the hallmark of motor adaptation, are commonly seen following adaptation to both visual and force perturbations in a number of tasks (Shadmehr and Mussa-Ivaldi, 1994; Scheidt et al., 2000; Krakauer et al., 2005; Smith et al., 2006). However, such after-effects can be substantially reduced through contextual cues that link the perturbation to the external world vs. the participant’s body (Lackner and Dizio, 2005; Kluzik et al., 2008). In other words, if a reliable external source of a perturbation is provided to the actor, they only adjust their motor plans when in that specific context. In comparison, if no such cues are available, the error is attributed to the self, and after-effects are observed.

Recent work has also shown that errors appear to be allocated with differing time scales. Specifically, using both visuomotor and force-field adaptation, two parallel processes have been identified. A fast process that adapts and de-adapts quickly, and an aptly named slow process that adapts and de-adapts with a more gradual time scale (Newell, 1991; Shadmehr and Mussa-Ivaldi, 1994; Krakauer et al., 2005; Smith et al., 2006; Huang and Shadmehr, 2009). The benefit of a system with two (or more) processes that vary in their temporal characteristics is that rapid learning mechanisms would enable the individual to deal with potentially short-lived perturbation (such as a gust of wind), and the slower mechanism(s) could be used in situations where the source of the error is longer lasting (such as fatigue). Of course, these timescales themselves must be flexible, with research showing that they can be adjusted depending on the rate of change previously experienced (Huang and Shadmehr, 2009).

To date, there have been very few studies examining issues of credit assignment during object lifting tasks, although it is apparent that object lifting also requires solutions to the credit assignment problem. To lift an object efficiently, one must predict the weight of the object to be lifted (Johansson and Westling, 1988; Wolpert and Flanagan, 2001; Flanagan et al., 2006; Johansson and Flanagan, 2009). An efficient lift can be described as one consisting of a smooth increase in vertical load force to a level that just exceeds the predicted weight of the object. When lifting a novel object, people are quite accurate at predicting its weight, provided it falls within our long-term size-weight (Gordon et al., 1991; Flanagan and Beltzner, 2000; Mon-Williams and Murray, 2000) or material-density priors (Gordon et al., 1993; Buckingham et al., 2009). Despite this proficiency in predicting object weight, there are times when these predictions will contain errors. In order to maximize future lifting performance, the source of an error related to an incorrect initial prediction of the forces required to lift an object off a surface should be identified by the motor system. For example, if the error in lifting performance was a result of interacting with an object with an unusual size-weight relationship, sensorimotor memory can be used to adapt future lifts of the same object (Johansson and Cole, 1992; Flanagan et al., 2006; Johansson and Flanagan, 2009), or a combination of sensorimotor memory and long-term priors can be used when extrapolating to newly encountered objects (Baugh et al., 2012). However, in all of these scenarios, errors in lifting must be correctly assigned to the external environment, or to the self, appropriately.

The current study was designed to examine how the rate of change in an object’s mass affects whether the experienced error in lifting performance are transferred to a novel to-be-lifted object. To address this issue, participants were asked to repeatedly lift a small cube. Unknown to the participant, the weight of that cube either increased from a weight of 400 g to a final weight of 570 g at a level below conscious perception over a series of 90 lifting trials, or suddenly increased from 400 g to the final weight midway through the experiment. After 90 lifts, all participants were then asked to lift a larger cube with an outer visual appearance that was different from the previously lifted blocks.

We hypothesized that if those participants in the gradual weight change condition interpreted the changes in object weight during the first 90 lifts to the self, they would lift the newly encountered larger block with greater lifting forces than those participants that were in the sudden weight change condition. Confirmation of this prediction would provide support for theories that posit the rate of change experienced plays a critical role in how the motor system solves the credit assignment problem.

## Materials and methods

### Participants

Thirty-nine participants (17 female; mean age 32; std. dev. 16) recruited from the University of South Dakota took part in this experiment after providing written informed consent. All participants performed the experiment with their dominant hand, as assessed by a modified Edinburgh handedness inventory (Oldfield, 1971). All experimental procedures were approved by the University of South Dakota’s Institutional Review Board, and participants received financial compensation (20 USD per hour) or course credit for their time. Participants were randomly assigned to one of two experimental conditions (see below).

### Apparatus

A total of 10 objects were used in this experiment. These included nine medium (216 cm^3^) sized objects of identical outer appearance, varying in weight from 400 g to 570 g. A lead core was added to the center of each object increasing the weight by 4% from the previous block weight, a value known to be below the just-noticeable-difference threshold (JND) for weight estimations in hand-held objects (Brodie and Ross, 1984; Jones, 1986; Pang et al., 1991). One large cube (729 cm^3^) was created with a different outer visual appearance (red vs. black), with a weight of 1354 g. The density of the small light-weight black cube (1.81 g/cm3) was chosen as it was unusually heavy for the apparent material (polylactic acid (PLA), 1.25 g/cm3) to ensure all participants were starting the experiment lifting a novel material-density relationship. The density of the heaviest black cube was 2.63 g/cm3, the resultant of the maximum change in mass possible within the JND threshold over the number of lifts participants performed. Finally, the density of the large red cube was also set to 1.81 g/cm3. As we have previously demonstrated (Baugh et al., 2012) when extrapolating to larger, unusually weighted blocks, weight predictions are brought down by the more stable long-term priors related to the apparent material. We anticipated that both groups (sudden and gradual) would predict that the large red block would have a lower density than the small black cube, as their estimates would be reduced by previous experiences with lifting plastic blocks. This allowed us to examine the differences in weight prediction between the two groups of participants, without having any participant over-estimate the weight of the block.

**Figure 1:**
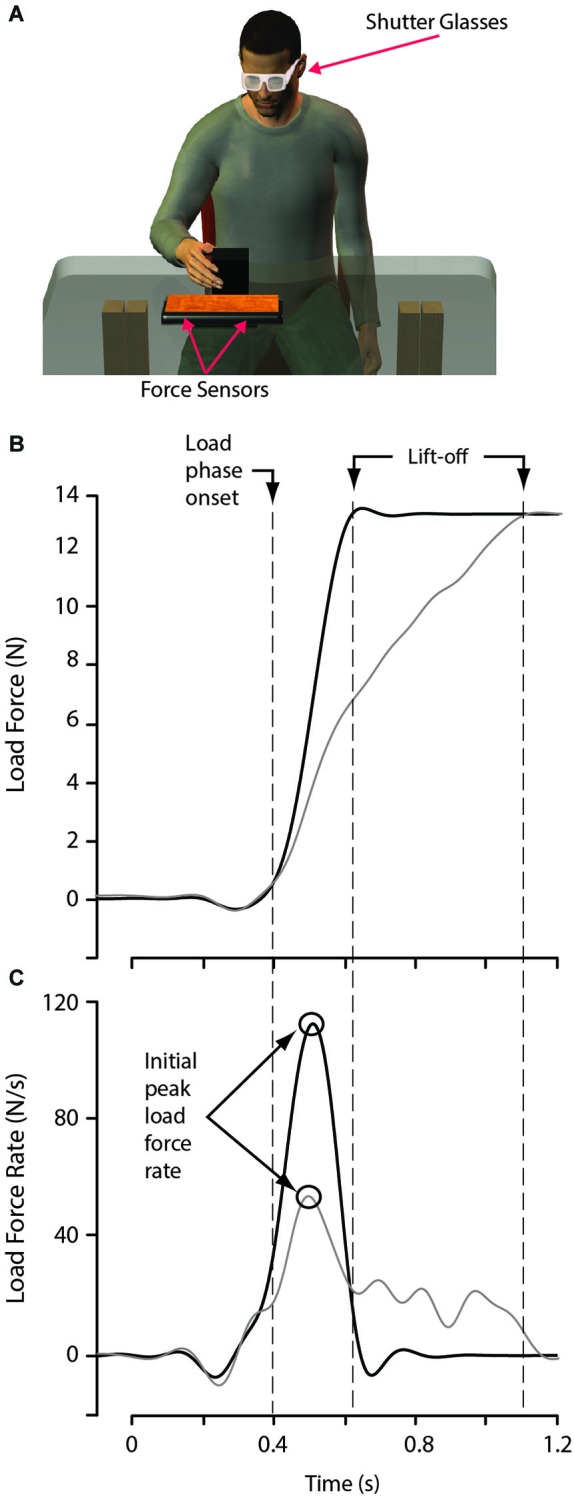
**Experimental apparatus and data analysis. (A)** While seated, participants lifted and replaced an object located on top of a platform instrumented with two force sensors. Shutter glasses were opaque between trials, preventing the participant from observing the to-be-lifted block being placed. **(B)** Load force function from two lifts of the large red object, in one lift (gray curves) the initial increase in load force was too low for the object weight, in the other lift (black curves), the initial increase in load force accurately reached the object weight. **(C)** Corresponding load force rate functions. Of importance, the initial peak in load force rate scaled with the initial increase in load force, which is dependent on predicted object weight.

During each trial, participants were required to lift an object from a tabletop platform (Figure 1A) instrumented with two force sensors (Nano 17 F/T sensors, ATI Industrial Automation, Garner, NC, USA) to a height of approximately 2.5 cm, hold the object stationary for 1 s and then place the object next to the platform. The force sensors were capped with a flat rectangular surface, with a width of 15 cm and a length of 26 cm. These force sensors allowed for the precise measurement of the vertical load force applied to the object during lifting, up to the point when the object lifted off the supporting platform. Participants wore LCD shutter-glasses (Plato Technologies, Toronto, Ontario, Canada) that blocked vision during the inter-trial intervals.

### Procedure

Participants were randomly assigned to one of two groups. One of the groups (Gradual, *N* = 20) lifted the entire range of 9 medium-sized objects 10 times in the training phase, with the object increasing to the next heavier weighted object every 10 lifts, for a total of 90 lifts. As all objects were identical in visual appearance, and the change in weight was below the JND threshold, participants were unaware of this change in object weight. The second group (Sudden, *N* = 19) completed the first half of the training phase (45 trials) with the lightest block and the second half of the training phase (45 trials) with the heaviest block. Following the 90 training lifts, both groups completed 10 lifts of the heavy red block.

The shutterglasses prevented participants from seeing the experimenter change the lifted object, and prevented any visual cues as to object weight. On all trials, the object was removed from the tabletop placed on a small table out of the participant’s view, and then was either replaced or returned to the lifting surface. As this procedure was identical in both trials in which the weight of the object was changed and those when the weight remained the same, no auditory cues were available to the participant to suggest the object had been replaced in either of the two conditions.

Participants received both verbal instructions and a demonstration by the experimenter as to how to perform the lifting motion. Participants were asked to lift the test object 2.5 cm (1″) off the sensor platform, hold it in mid-air for 1 s, and then place it on the tabletop. An auditory tone (500 Hz, 1 s) indicated when the participant was to begin the lift, and coincided with the shutterglasses turning translucent. A second tone (250 Hz, 1 s) indicated when the participant was supposed to place the object back on the tabletop. At the end of each trial, the shutter glasses turned opaque, preventing vision during the inter-trial interval.

### Data analysis

Vertical forces from the sensors were sampled at 250 Hz. Raw force signals were low-pass filtered using a 4th order, zero-phase lag Butterworth filter with a cut-off frequency of 14 Hz, offline. A signal representing the vertical force applied to the object by the hand (i.e., the vertical load force) was obtained by subtracting the initial vertical force accounting for the weight of the object when fully supported by the lifting platform from the recorded signal. This processed signal was then differentiated with respect to time using a 1st order central difference equation, resulting in the rate of change in the load force.

For each lift of the test objects, the first peak in load force rate and the load force associated with this peak were determined. The start of the load phase was defined as the time point in which the load force first exceeded 0.2 N (Figure 1B). Therefore, the first peak in load force rate (defined as a maxima followed by a decrease) had to occur after the load force exceeded 0.2 N. This threshold of 0.2 N was selected as load force values earlier in the trial are likely the result of initial finger placement on the block, rather than an obvious attempt to lift the object (Figure 1C). The end of the load phase was defined as the time, just before object lift-off, when load force reached within 0.2 N of the weight of the object (Figure 1B). Due to objects being lifted off the force-sensing platform, recording load forces after lift-off was not possible. When assessing initial predictions of object weight, such a method is adequate because the initial peak rate of change of load force occurred well before object lift-off in all of the trials examined.

When lifting objects, people tend to normalize the lift duration across object weight by scaling the load force rate, prior to object lift off, to the expected weight of the object. Further, by using a small target lift height, participants typically will reduce the load force rate so that it approaches zero at the expected lift-off time. Due to these task characteristics, the peak rate of change of load force during the initial increase in load force and the load force at the peak rate of change in load force rate are accurate reflections of the participants predicted weight of the object (Johansson and Westling, 1988; Flanagan and Beltzner, 2000; Flanagan et al., 2008).

In many object lifting studies, both vertical load forces and horizontal grip forces have been measured. This is typically accomplished by having participants lift an object via a handle instrumented with force sensors. In the present study, participants lifted the object directly off the force sensors, preventing the collection of grip force data. Justifying this approach, load force provides a more accurate measure of the participant’s expected weight than grip force, because load force depends solely on object weight, whereas grip force depends on object properties not directly related to the mass, such as friction between the object and digits (Westling and Johansson, 1984). A primary advantage of the method utilized in this study is that participants directly manipulate the object, and therefore obtain a more natural lifting experience (Flanagan et al., 2008).

Data analysis focused on the first lift of the black cube as this reflects a participant’s initial predictions as to the weight of the object. The last lift of the black cube was also examined, to establish no differences existed between our groups before changing to the novel large block, as these should be equivalent between the two groups of participants as they are lifting the same 547 g block immediately preceding the switch to the large block. Of critical importance were the first three lifts of the newly encountered large red block. This block was weighted to be unusually heavy for its size and apparent material at 1354 g, which allowed us to examine any differences the rate of change experienced in the previous lifting trials had on the initial weight predictions of the novel block. Following the experiment proper, participants in both the gradual and sudden groups were debriefed as to the true nature of the experiment.

## Results

No participants within the gradual group reported sensing the object weight change during debriefing. The initial peak load force rate (PeakLFR),the load force at the initial peak load force (LF@PeakLFR) rate and the load phase duration (LPD) were submitted to a 3 (lift—first 3 lifts of the small black blocks, last 3 lifts of the small black block, and first 3 lifts of the large red block) × 2 (group—Gradual vs. Sudden) repeated-measures analysis of variance (rm-ANOVA) with lift as a within-subject factor and condition as a between subject factor. For all three measures (PeakLFR aLF@PeakLFR and LPD), a significant interaction between Lift and Condition was observed (*F*_(2,74)_ = 5.435, *P* = 0.006; *F*_(2,74)_ = 3.763, *P* = 0.028; *F*_(2,74)_ = 4.395, *P* = 0.016, respectively), demonstrating the effect of lift was not consistent across our two groups of participants. There was also a main effect of lift, demonstrating that all participants adjusted their lifting forces to the weight as the presented block changed. Specifically, the heavier blocks used in later trials were lifted with greater forces when compared to the lighter blocks used in the earlier trials (*F*_(2,74)_ = 177.305, *P* < 0.001; *F*_(2,74)_ = 216.642, *P* < 0.001), and were associated with shorter lift durations (*F*_(2,74)_ = 39.92, *P* < 0.001).

**Figure 2:**
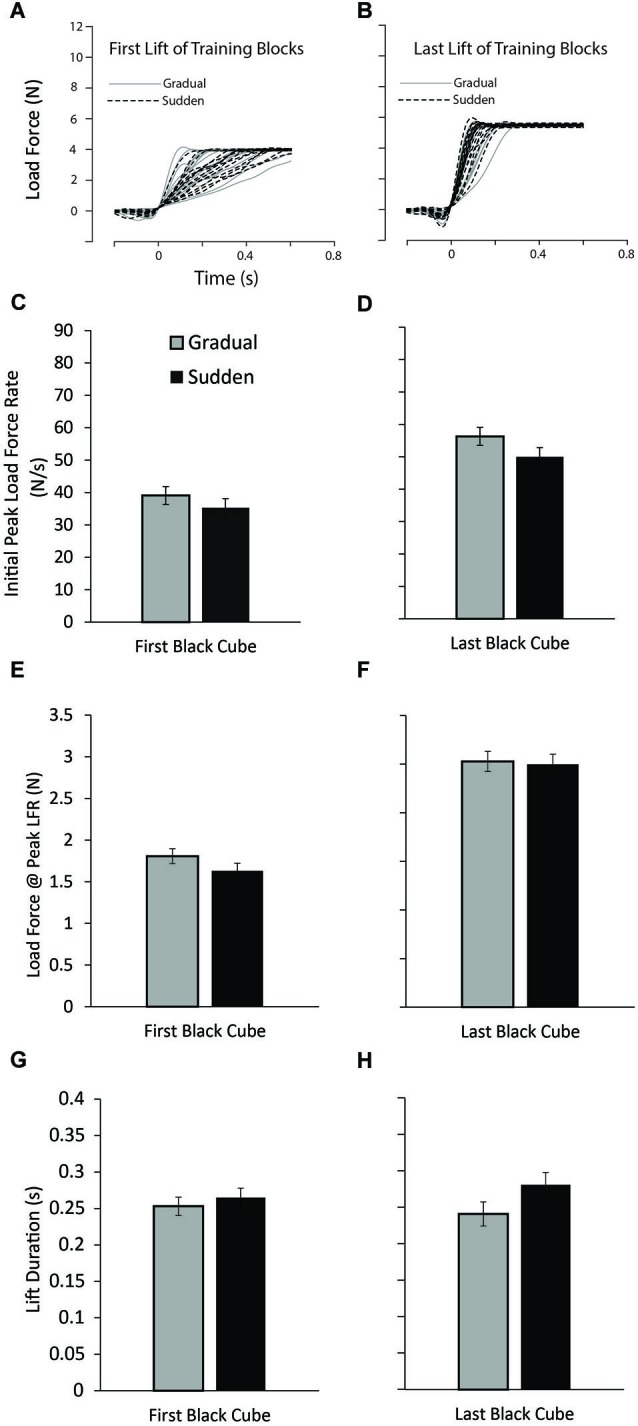
**Lift performance on the training blocks. (A)** Load force records as a function of time from the first lift of the training block for all participants in both the sudden (black dashed line) and gradual (solid gray line) lifting conditions. **(B)** Load force records for the last lift of the training block for all participants. **(C–H)** Mean initial peak load force rate (PeakLFR) (**C** and **D**), load force at initial peak in load force rate (**E** and **F**), and load phase durations (LPD) (**G** and **H**) averaged across participants, for the first (**C**, **E**, and **G**) and last (**D**, **F**, **H**) of the lifts of the training blocks. Vertical lines represent ±1 standard error.

We expected that, following repeated lifting of the small black cubes, participants in all groups would learn to adequately predict the forces required to efficiently lift the object, as indicated by force output appropriately scaled to the actual weight of the object. To ensure this was the case, we examined the first and last lifts of the small black cubes, as these were equivalent in weight for both the Gradual and Sudden lifting groups. Figure 2 shows that participants in both groups began the experiment with approximately equal load forces when lifting the initial training blocks (A) and efficiently increased load force up to the weight of the object, and showed no differences in lifting forces in the last lifts of the training block (B). Additionally, planned comparisons between conditions on the first lift of the training block and at the last lift of the training block revealed no differences in the PeakLFR (*t*_(37)_ = 0.961, *P* = 0.343; *t*_(37)_ = 1.294, *P* = 0.204, respectively), LF@PeakLFR (*t*_(37)_ = 1.362, *P* = 0.181; *t*_(37)_ = 0.214, *P* = 0.832, respectively), or LPD (*t*_(37)_ = −0.661, *P* = 0.513; *t*_(37)_ = −1.691, *P* = 0.099) (C and D; E and F; G and H ). These results demonstrate both groups of participants began the experiment without significant differences in lifting forces or lift durations and ended the training phase of the experiment without significant differences in lifting forces or lift durations.

**Figure 3:**
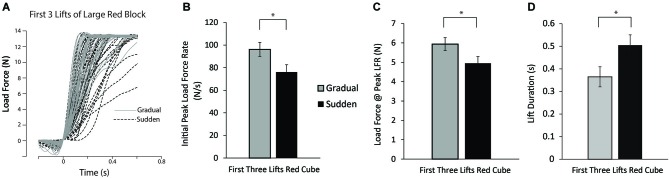
**Lift performance on first lifts of the large red block. (A)** Load force records as a function of time from the first three lifts of the large red block for all participants in both the sudden (black dashed line) and gradual (solid gray line) lifting conditions. **(B–D)** Mean initial PeakLFR **(B)**, load force at initial peak in load force rate **(C)**, and LPD **(D)** averaged across participants. Vertical lines represent ±1 standard error, asterisk denotes statistical significance at the *p* < 0.05 level.

Load force tracings of the first three lifts of the large red block for all participants can be seen in Figure 3A. To test our hypothesis that those participants in the gradual weight change group would lift the newly encountered red blocks with greater force than those participants in the sudden weight change condition, planned comparisons between the first three lifts of the large red block between each group were performed. Significant differences in PeakLFR, LF@PeakLFR, and LPD was found (*t*_(37)_ = 2.223, *P* = 0.032; *t*_(37)_ = 2.080, *P* = 0.044, respectively) (Figures 3B–D).

**Figure 4:**
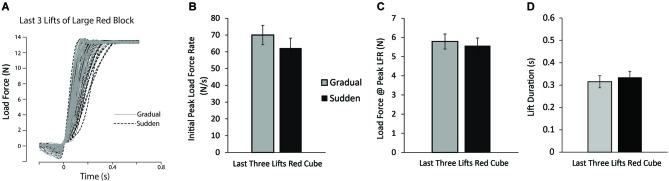
**Lift performance on last lifts of large red block. (A)** Load force records as a function of time from the last three lifts of the large red block for all participants in both the sudden (black dashed line) and gradual (solid gray line) lifting conditions. **(B–D)** Mean initial PeakLFR **(B)**, load force at initial peak in load force rate **(C)**, and LPD **(D)** averaged across participants. Vertical lines represent ±1 standard error.

Finally, to examine the longevity of this effect, planned comparisons between the last three lifts of the large red block for the gradual and sudden groups were performed. Load force tracings of the last three lifts of the large red block for all participants can be seen in Figure 4A. No significant differences in PeakLFR or LF@PeaklLFR were found (*p’*s > 0.10) (Figures 4B–D), suggesting the observed effect was short-lived.

## Discussion

Although temporal credit assignment has been examined in a number of different scenarios, little to no research has examined these issues during natural object lifting tasks, despite such scenarios also requiring a solution to the credit assignment problem. Specifically, in order to maximize future lifting performance with an object, the ability to accurately predict the forces necessary to lift said object is an essential component of dexterous object manipulation (Johansson and Westling, 1988; Johansson and Flanagan, 1999; Wolpert and Flanagan, 2001; Flanagan et al., 2006), and the temporal nature of the errors applied is likely to be an important factor within this prediction, as has been demonstrated in other tasks. This study is the first to our knowledge that demonstrates experience with an object lifting task is also sensitive to the rates of change in object weight. The role of the temporal nature of changes in object weight was influential in how the errors experienced during object lifting were applied to a novel lifting scenario. Further, we believe how the experienced errors were credited are related to whether the perturbation was seen as arising from the self or from the external environment. As we had predicted, we found the rate of change in object weight that the participants experienced had a strong influence on participants’ weight predictions when encountering a newly presented object. Specifically, we demonstrated that those participants that experienced a scenario in which object weight was slowly increased lifted a newly encountered object with a greater initial peak in load force rate, and a greater load force at the initial peak in load force rate than those participants who experienced a faster change in object weight in the training phase. When participants were required associate a greater weight with the object to be lifted, they were required to link this learning to appropriate contextual cues for it to be used in later interactions. Due to participants lifting objects off force sensors, we chose to make the order of weight change always go from lighter to heavier in both the gradual and sudden conditions. This effectively ensured that a participant’s predicted weight of the test object was either equal to or less than the actual weight, allowing accurate load force measurements to be obtained from the sensor before object lift-off occurred. However, had we made the order reversed (going from heavier to lighter), we would expect that rates of change in object weight would have the same effect as what we observed in the present study.

Recent models of temporal credit assignment provide a possible mechanism by which this linking may occur (Smith et al., 2006; Lee and Schweighofer, 2009). Under these models, fast and slow learning processes act in parallel in response to error signals, but differ in both their rates of learning and unlearning. As the name would suggest, slow learning processes are slower to adapt but also have a slower decay rate. In contrast, the fast system is quick to adapt and to de-adapt. In support of these models, research has shown that learning in a rapidly changing environment is affected by the temporal features of the task. Specifically, when participants made reaching movements in an environment which contained rapid changes, the decay rate of motor memories was greater than when participants were exposed to an environment with gradual changes (Huang and Shadmehr, 2009).

When examining the magnitude of the effect reported here, it is interesting to note that the increase in load forces utilized in lifting the novel large red cube is consistent with the weight change experienced by the gradual participant group during the training period. An efficient object lift typically consists of unimodal, bell-shaped distribution when examining the rate of change in load force. Therefore, the initial peak in load force is scaled to the predicted object mass, with the load force at the initial peak in load force rate being approximately half of the predicted object mass (Johansson and Westling, 1984, 1988). When comparing the LF@PeakLFR between the gradual and sudden participant groups, we observed a difference of approximately 1 N, which is quite close to the 0.83 N that one would expect based on this simple relationship between load forces and predicted object weight.

It is important to note that we are not claiming that the motor system is unable to adapt to the increasing object weight when participants were exposed to a gradual change in object weight. In fact, when examining the lifting forces utilized at the end of the training session, those participants in the gradual and sudden weight change condition were applying equal lifting forces appropriately matched to the actual object weight (see Figure 2). Additionally, when examining the longevity of this effect, after 10 lifts of the large red cube differences between our groups in any of our measures were not present. This suggests that even though there were differences between our two experimental groups, these differences were short-lived and in both the gradual and sudden change participant groups, lifting forces were appropriately scaled for the object to be lifted. This is in congruence with previous studies showing that when lifting objects with poorly predicted weight, the motor system adapts to the actual object weight within approximately 10 trials (Flanagan and Beltzner, 2000; Grandy and Westwood, 2006; Flanagan et al., 2008).

In the present study, we did not directly assess which features of the gradual and sudden weight change conditions are used by the motor system to determine generalizability. For instance, in the sudden condition, in addition to differences in the temporal dynamics, the size of the error subjects experienced is much larger than the error experienced in the gradual condition. That is to say, that the difference between the predicted forces necessary to lift the object, and the eventual force required on a trial in which the object changed weight was much greater than the difference experienced by those participants in the gradual weight change condition. There is mounting evidence that suggests small errors affect learning in a fundamentally different way when compared to large errors (Criscimagna-Hemminger et al., 2003; Malfait and Ostry, 2004; Hatada et al., 2006; Michel et al., 2007; Huang and Shadmehr, 2009), and there is some evidence to support the neurological correlates related to error correction in these two scenarios is distinct (Criscimagna-Hemminger et al., 2010). Another important distinction between our rapid and gradual adaptation conditions is whether the subject is cognitively aware of the error. Our gradual condition was designed so that participants were unaware of the change in object weight over time, in contrast to those participants in the sudden condition. Previous adaptation work has shown that whether a participant is aware of a perturbation can change generalization patterns (Malfait and Ostry, 2004), and in some cases result in improved performance in a reaching task (Hwang et al., 2006), and in others result in decreased performance (Mazzoni and Krakauer, 2006).

Although we believe the presented results provides evidence that fast rates of change in an object lifting task are attributed to external sources of error, whereas slow rates of change are attributed to internal sources (in agreement with previous reaching work) due to the increased complexity during skilled object manipulation a number of alternative explanations warrant discussion. Firstly, it is possible that those participants in the gradual weight change condition could adjust their internal representation of object density—an external attribution. We would predict if such a process were responsible for the differences between our two groups, we would have observed much higher load forces during the first three lifts of the large red block. Extrapolating from the final density of the small black cube, the predicted weight of the large red cube would be approximately 2000 g. In opposition to this, the magnitude of the observed effect was much smaller than this value. A second alternative hypothesis could be that participants in the sudden weight change condition developed an average sensorimotor memory of object density that was utilized when extrapolating lifting forces to the large red cube. We are unaware of studies which show such an effect, and most research has demonstrated that during conditions of unpredictable object weight changes, load forces are largely correlated with the immediately preceding lift (Johansson and Westling, 1988; Forssberg et al., 1992; Gordon et al., 1994; Salimi et al., 2000). Nevertheless, as the present study was not directly designed to rule out such alternatives, some questions as to the root source of the differences in load forces between the sudden and gradual participant groups. Future research will examine the distinct roles each of these features play when the motor system attempts to assign error to motor predictions in object lifting tasks.

The presented research helps to move the examination of motor learning away from a fairly limited number of scenarios tested in the laboratory (such as reaching under perturbation) and into the more complicated realm of real-world motor control. In our day-to-day lives, we are often presented with objects that may adjust in mass with or without our knowledge, and the ability for the brain to be able to correctly attribute errors in prediction under these circumstances is critical for dexterous manipulation of our surroundings. Bilateral hemispheres and the right vermis of the cerebellum are known to become active during object lifting (Kinoshita et al., 2000; Schmitz et al., 2005) and cerebellar damage can result in precision grip deficits, especially in the coordination of grip force and load force during perturbation (Müller and Dichgans, 1994; Babin-Ratté et al., 1999; Serrien and Wiesendanger, 1999; Fellows et al., 2001; Rost et al., 2005). These results are consistent with theories that posit an internal model related to limb dynamics is implemented within the cerebellum (Wolpert et al., 1998; Blakemore et al., 2001; Wolpert and Flanagan, 2001; Kawato et al., 2003). In addtion, it has been demonstrated that the cerebellum plays a crucial role in the fast learning system and that patients with cerebellar damage may show deficits in the fast component of motor learning (Morton and Bastian, 2004, 2006; Diedrichsen et al., 2005; Smith and Shadmehr, 2005; Tseng et al., 2007). Additionally, transcranial direct current stimulation of the cerebellum can increase the rate of adaptation to a sudden perturbation, and primary motor cortex stimulation can improve retention of the perturbation (Galea et al., 2011), verifying that the cortico-cerebellar loop is involved in the formation and retention of learned adaptation. There is also mounting evidence that the fast component of motor learning shares critical resources with declarative memory, and is subject to interference affects during dual-task paradigms (Anguera et al., 2010; Keisler and Shadmehr, 2010). Much less is known about the slow component of motor learning, aside from the fact that it is likely a distinct process and may be related to the same anatomical substrates as procedural memory (Keisler and Shadmehr, 2010). Future research would be well-served by attempting to further dissociate these two timescales of motor learning at the neuronal level.

## Conflict of interest statement

The authors declare that the research was conducted in the absence of any commercial or financial relationships that could be construed as a potential conflict of interest.
